# Plasminogen activator inhibitor-1 activity and long-term outcome in patients with ST-elevation myocardial infarction treated with primary percutaneous coronary intervention: a prospective cohort study

**DOI:** 10.3325/cmj.2018.59.108

**Published:** 2018-06

**Authors:** Marin Pavlov, Vjeran Nikolić-Heitzler, Zdravko Babić, Milan Milošević, Krešimir Kordić, Ivana Ćelap, Vesna Degoricija

**Affiliations:** 1 Department of Cardiology, Sestre Milosrdnice University Hospital Center, Zagreb, Croatia; 2 University of Zagreb School of Medicine, Zagreb, Croatia; 3 University of Zagreb School of Medicine, Andrija Štampar School of Public Health, Zagreb, Croatia; 4 Department of Clinical Chemistry, Sestre Milosrdnice University Hospital Center, Zagreb, Croatia; 5 Department of Medicine, Sestre Milosrdnice University Hospital Center, Zagreb, Croatia

## Abstract

**Aim:**

To determine the relationship between plasminogen activator inhibitor-1 (PAI-1) activity rise during the first 24 hours of ST-elevation myocardial infarction (STEMI) treatment and death after 5 years.

**Methods:**

From May 1, 2009 to March 23, 2010, 87 STEMI patients treated with primary percutaneous coronary intervention (PCI) at the Sestre Milosrdnice University Hospital Center were consecutively enrolled in prospective single-center cohort study. PAI-1 activity was determined on admission and 24 hours later. The primary end-point was death after 5 years. The predictive value of PAI-1 activity variables as biomarkers of death was assessed using receiver operating characteristic (ROC) curve, independent predictors of death were assessed using multivariate Cox regression, and covariates independently related to higher PAI-1 activity rise were assessed using linear regression.

**Results:**

Two patients died during the hospital treatment and 11 during the follow-up. PAI-1 activity rise had the largest area under curve (0.748) for predicting death rate (optimal cut-off point 3.7 U/mL, sensitivity 53.8%, specificity 90.5%). Patients with PAI-1 activity rise higher than 3.7 U/mL had significantly higher mortality (*P* < 0.001). Kaplan-Meier survival curve diverged within the first year after STEMI. Independent predictors of death were PAI-1 rise and final TIMI flow. PAI-1 activity rise was independently related to heart failure, thrombus aspiration, and body weight.

**Conclusion:**

PAI-1 activity rise higher than 3.7 U/mL is associated with higher 5-year death rate in STEMI patients treated with primary PCI.

Oxford Centre for Evidence-based Medicine level of evidence: 3.

Myocardial infarction (MI) is initiated by a coronary artery atherosclerotic plaque rupture or erosion, followed by a thrombotic event leading to a complete or partial occlusion (1). Thrombus stabilization and extension or spontaneous reperfusion depend on the interplay of endogenous prothrombotic and fibrinolytic factors (2). One of such factors is plasminogen activator inhibitor-1 (PAI-1). It is the central inhibitor of plasminogen activation, whose function is to deter endogenous fibrinolysis (3,4). PAI-1 is predominantly secreted by platelets and endothelial cells and is present in plasma in an active, inactive, and latent form (5). Its distinct circadian pattern, with the highest concentration and activity in the morning, has to be taken into account in studies using single-sample values (6).

PAI-1 role in acute MI has been studied only regarding short-term outcomes. Collet et al (7) reported the predictive value of PAI-1 antigen level rise (measured at patient presentation and after 24 hours to avoid the influence of circadian variations) and the final flow according to the Thrombolysis in Myocardial Infarction (TIMI) criteria for 30-day mortality of ST-elevation myocardial infarction (STEMI) patients. However, that study included both acute and subacute MI patients and had a penetration rate of urgent invasive strategies of 77.1%. Also, the majority of the deaths (9 of 11) occurred during hospital treatment. There are no available data on the predictive value of PAI-1 rise for the long-term outcome in STEMI patients treated with primary percutaneous coronary intervention (PCI). We hypothesized that PAI-1 activity rise in these patients was related to poorer long-term outcome. The aim of the study was to determine the relationship between PAI-1 activity rise and long-term outcome and to examine clinical, angiographic, and laboratory parameters related to higher rise in PAI-1 activity.

## Patients and methods

### Patients

One hundred consecutive acute STEMI patients treated with primary PCI within 12 hours from the symptoms onset at the Sestre Milosrdnice University Hospital Center from May 1, 2009 to March 23, 2010 were examined for eligibility to enter this prospective single-center cohort study. The only inclusion criterion was STEMI diagnosis, established if persistent ST-segment elevation ≥1 mm in at least 2 consecutive standard electrocardiogram (ECG) leads or persistent ST-segment elevation ≥2 mm in precordial ECG leads, or new onset complete left bundle branch block, was observed and accompanied by chest pain lasting more than 20 minutes. Inability or refusal to sign the informed consent was regarded as a non-inclusion criterion. Ethical approval was received from Sestre Milosrdnice Clinical Hospital Center Ethics Committee.

The exclusion criteria were as follows: not available or deceased at 24 hours (1 patient), chronic corticosteroid or immunosuppressive therapy (4 patients), no significant coronary artery disease on angiogram (2 patients), diagnosis of acute infective or inflammatory disease established within the first 24 hours (5 patients), active malignant disease (1 patient), and chronic hepatic failure requiring treatment (0 patients). A total of 87 patients entered the study. The number of participants was restricted by available financial resources, which were used for laboratory analyses.

### Methods

Blood samples were collected in citrated tubes on admission (before PCI) in the catheterization laboratory and exactly 24 hours later in the Cardiac Intensive Care Unit. The samples were centrifuged for 10 minutes at 4000 rotations per minute, frozen at -20°C, and analyzed for PAI-1 activity after no more than 28 days (8).

PAI-1 activity was analyzed using the commercially available Berichrom PAI on BCS XP analyzer (both from Siemens, Marburg, Germany) (9). After STEMI diagnosis was established, all patients received a routine acute-phase STEMI treatment, consisting of a loading dose of 300-mg aspirin and 600-mg clopidogrel. Patients with STEMI transferred from county hospitals in Karlovac (53 km) and Sisak (59 km) and patients presenting at the emergency department of Sestre Milosrdnice University Hospital Center and diagnosed with STEMI were immediately admitted to the catheterization laboratory. All PCIs were performed using transfemoral approach. The attending cardiologist decided on the dose and indication for the use of heparin, eptifibatide, thrombaspiration, type of intervention, and materials. The initial and final coronary flow were determined according to standard TIMI flow classification (10). No-reflow phenomenon was diagnosed if post-PCI residual culprit lesion stenosis was <50% and final TIMI flow was <3. After the procedure, patients were administered medications, including dual antiplatelet therapy, beta blockers, angiotensin converting enzyme (ACE) inhibitors, and statins according to indications and contraindications, by the attending cardiologist. Medical history, clinical parameters (occurrence of heart failure, cardiogenic shock, arrhythmias, and death), and laboratory findings were recorded prospectively. Creatine kinase (CK) peak level was determined as the highest value among the consecutive 6-hour findings; the analysis was stopped after the first value lower than the previous one was observed. Echocardiography was performed during the hospital stay. Primary end-point was defined as death after 5 years. The outcome data were obtained from the medical records and by a telephone interview (with family members for deceased patients) at a fixed time point of 5 years after the initial hospitalization.

### Statistical analysis

Depending on the normality of distribution, tested by Kolmogorov-Smirnov test, descriptive data are presented as means and standard deviations (SD) or medians and interquartile ranges (IQR). Categorical variables are presented as counts and frequencies. The difference between PAI-1 activity values on admission and after 24 hours was tested using Wilcoxon signed-rank test. PAI-1 activity rise was defined as the value on admission subtracted from the value obtained 24 hours later. Categorical variables were analyzed with χ^2^, Fisher exact, or Fisher-Halton-Freeman exact test, continuous by Mann-Whitney U test, and correlations by point-biserial correlation. Receiver operating characteristic (ROC) curves were used to determine variable’s predictive value and optimal cut-off points (using Youden index). Binary logistic and linear regression models were used to determine independent contribution of an individual variable to PAI-1 activity variability. Survival was analyzed using Cox regression and log-rank test, and presented as Kaplan-Meier curves. Two-tailed significance tests were performed, and *P* < 0.05 was considered significant. Statistical analyses were performed using SPSS for Windows, version 25 (IBM SPSS, Armonk, NY, USA, licensed to the University of Zagreb, School of Medicine).

## Results

**Figure 1:**
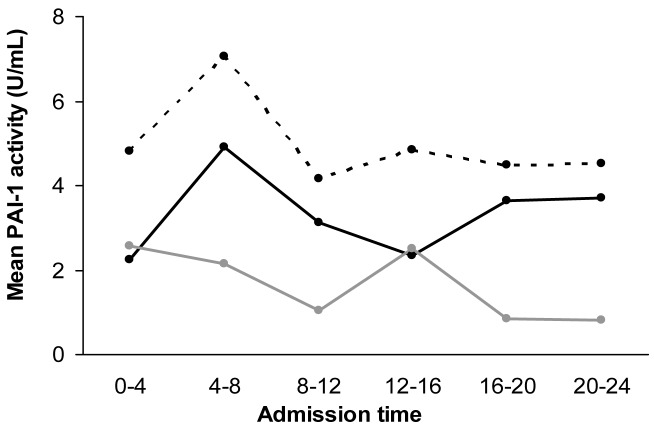
Mean plasminogen activator inhibitor-1 (PAI-1) activity levels on admission and 24 hours later according to the time of day when the samples were obtained. Full black line – PAI-1 activity on admission; dotted black line – PAI-1 activity after 24 hours; full gray line – PAI-1 activity rise.

Study population was predominantly male, aged 61.1 ± 12.2 years, with a high prevalence of patients with hypertension and active smokers (Table 1). Low admission troponin T levels and high peak CK levels were observed, while all patients had initial TIMI flow lower than 3. High penetration of balloon angioplasty and frequent use of eptifibatide was observed, and 95.4% of interventions ended with stent implantation. Both PAI-1 measurements were obtained for all 87 patients. The highest mean PAI-1 activity levels in both samples were detected early in the morning (Figure 1); however, PAI-1 activity rise did not differ significantly among 4-hour time intervals of the day. PAI-1 activity measured after 24 hours was significantly higher than that on admission (4.71 ± 2.35 vs 3.25 ± 1.99 U/mL; *P* < 0.001, Wilcoxon signed-rank test). PAI-1 activity rise correlated with heart failure occurrence (r_pb_ = 0.393, *P* < 0.001), thrombus aspiration (r_pb_ = 0.344, *P* = 0.001), pulmonary edema (r_pb_ = 0.267, *P* = 0.012), and final TIMI flow lower than 3 (r_pb_ = 0.235, *P* = 0.029).

**Table 1:** Characteristics of patients with ST-elevation myocardial infarction (STEMI) for total study sample and according to 3.7 U/mL cut-off value of plasminogen activator inhibitor-1 (PAI-1) activity increase*

Characteristic	No. of STEMI patients				
	Total (n = 87)	PAI-1 rise >3.7 U/mL (n = 14)		PAI-1 rise ≤3.7 U/mL (n = 73)	*P*
Age (years; mean±SD)	61.1 ± 12.2	66.2 ± 13.0		60.1 ± 11.9	0.104
Female sex	25	8		17	0.010
Transferred patients	33	7		26	0.310
Hypertension	58	8		50	0.409
Diabetes	14	1		13	0.451
Smoking	42	5		37	0.305
Previous coronary artery disease	11	2		9	1.000
Chronic medications:					
aspirin	13/86	4	9		0.213
beta blockers	12/83	3	9		0.417
ACE inhibitors or ARB	20/83	5	15		0.265
statins	6/86	0	6		0.583
Pain-to-first medical contact (minutes; median, IQR) [n]	122 (60-250) [86]^†^	188 (57-300) [14]	120 (61-247.5) [72]		0.884
First medical contact-to-balloon (minutes; median, IQR) [n]	60 (48.75-85) [86]^†^	60 (48-91.5) [14]	59.5 (48.25-84.75) [72]		0.893
Occurrence of heart failure	9	5	4		0.005
Pulmonary edema	1	1	0		0.161
Cardiogenic shock	3	2	1		0.066
Ventricular fibrillation^‡^	11	4	7		0.072
New onset atrial fibrillation	4	0	4		1.000
Anterior wall myocardial infarction^§^	34	9	25		0.035
Infarction-related artery					
Left anterior descending coronary artery	36	10	26		0.048
Circumflex coronary artery	10	1	9		
Right coronary artery	41	3	38		
Two- or three-vessel disease	51	7	44		0.475
In-stent thrombosis	2	1	1		0.298
Initial/final TIMI flow					
0	66/1	12/1	54/0		initial flow 0.882, final flow 0.033
1	12/1	1/1	11/0		
2	9/10	1/2	8/8		
3	0/75	0/10	0/65		
No-reflow phenomenon	23	7	16		0.029
Balloon angioplasty	66	10	56		0.736
Stent implantation	83	13	70		0.511
Use of drug-eluting stent (number of patients)^‖^	3/83	1	2		0.404
Thrombaspiration	12	5	7		0.009
Eptifibatide	63	10	53		1.000
Medications within first 24 hours:					
beta blockers	47	8	39		0.798
ACE inhibitors or ARB	38	4	34		0.252
statins	48	7	41		0.772
Unfractionated heparin/kg (U/kg; median, IQR) [n] ^¶^	85.4 (71.2-100.0) [86]^†^	94.2 (74.4-112.5) [14]	84.7 (70.6-100.0) [72]		0.165
C-reactive protein (U/L; median, IQR) [n]	2.9 (1.6-5.7)	2.1 (0.9-3.5) [14]	3.1 (1.6-6.8) [73]		0.097
Troponin T (μg/L; median, IQR) [n]	0.083 (0.024-0.199) [64]^†^	0.142 (0.036-0.243) [8]	0.083 (0.022-0.163) [56]		0.549
Peak creatine kinase (U/L; median, IQR) [n]	2639 (1480-4435)	4354 (2560-7387) [14]	2566 (1415-4075) [73]		0.010
Total cholesterol (mmol/L; mean±SD) [n]	5.93 ± 1.29 [81]^†^	6.09 ± 0.88 [13]	5.90 ± 1.36 [68]		0.463
Low density lipoprotein (mmol/L; mean±SD) [n]	3.91 ± 1.02 [81]^†^	4.02 ± 0.59 [13]	3.89 ± 1.09 [68]		0.593
High density lipoprotein (mmol/L; median, IQR) [n]	1.10 (1.00-1.30) [81]^†^	1.30 (1.15-1.50) [13]	1.10 (0.92-1.30) [68]		0.007
Triglycerides (mmol/L; median, IQR) [n]	1.80 (1.10-2.40) [81]^†^	1.70 (0.95-2.20) [13]	1.85 (1.20-2.47) [68]		0.321
Creatinine clearance <30 mL/kg	8/86	3	5		0.118
Left ventricular ejection fraction (%; median, IQR) [n]	50 (45-56) [67]^†^	51 (34-57) [10]	50 (47-56) [57]		0.441
Weight (kg; mean±SD) [n]	83.0 ± 14.9 [86]^†^	74.1 ± 11.5 [14]	84.7 ± 14.9 [72]		0.007
Body mass index (kg/m^2^; mean±SD) [n]	27.7 ± 4.0 [86]^†^	26.5 ± 3.7 [14]	27.9 ± 4.0 [72]		0.066
Waist circumference (cm; mean±SD) [n]	99.2 ± 13 [86]^†^	95.4 ± 13.8 [14]	99.9 ± 13.4 [72]		0.109
Hip circumference (cm; median, IQR) [n]	98.5 (94-105) [86]^†^	97.5 (91.5-100.7) [14]	100.0 (95.0-107.5) [72]		0.129
Waist-to-hip (ratio; median, IQR) [n]	0.990 (0.929-1.041) [86]^†^	0.978 (0.926-1.027) [14]	1.014 (0.930-1.043) [72]		0.390

*ACE – angiotensin converting enzyme; ARB – angiotensin receptor blocker; IQR – interquartile range; PAI-1 – plasminogen activator inhibitor-1; SD – standard deviation; TIMI – thrombolysis in myocardial infarction.

†Patients with incomplete data were omitted.

‡In 9 patients before and in 2 patients (within 6 hours) after percutaneous coronary intervention.

§According to electrocardiogram.

‖Out of all patients to whom stents were implanted.

¶During percutaneous coronary intervention.

PAI-1 rise was then expressed as a binary variable with the cut-off point of 3.7 U/mL, as determined by ROC analysis of long-term survival. Variables related to PAI-1 activity rise higher than 3.7 U/mL were heart failure, anterior wall myocardial infarction, left anterior descending artery as an infarction-related artery, use of thrombus aspiration, no-reflow phenomenon, worse final TIMI flow, and female sex among categorical variables (Table 1), and higher levels of peak CK, high-density lipoprotein, and lower body weight among continuous variables. The two PAI-1 activity rise groups did not differ significantly in therapy use (dose of heparin and use of eptifibatide during PCI, use of beta blockers, ACE inhibitors, and statins within first 24 hours).

Stepwise binary logistic regression included 7 variables significant in the univariate analysis (high-density lipoprotein was omitted due to a number of missing values, and TIMI flow and affected artery due to the lowest significance in the univariate analysis). It showed that independent predictors of PAI-1 rise higher than 3.7 U/mL were heart failure (exponentiation of b [e^b^] = 35.3; *P* = 0.004), thrombus aspiration (e^b^ = 15.2; *P* = 0.008), female sex (e^b^ = 5.0; *P* = 0.043), body weight (e^b^ = 0.936; *P* < 0.001), and peak CK levels (e^b^ = 1.0004; *P* = 0.013). Stepwise linear regression with PAI-1 rise expressed as a continuous variable showed that independent predictors of higher PAI-1 rise were heart failure (odds ratio [OR] 4.4, 95% confidence interval [CI] 1.8-4.8; *P* < 0.001), thrombus aspiration (OR 3.8, 95% CI 1.2-3.9; *P* < 0.001), and body weight (OR -2.2, 95% CI -0.06- to -0.01; *P* = 0.034).

Two patients (2.3%) died during hospital treatment, both due to acute complications of myocardial infarction. All discharged patients were available for 5-year follow-up. Eleven additional deaths (12.6%) occurred during follow-up due to the following causes: cardiac causes in 8 patients (3 sudden cardiac deaths, 4 recurrent myocardial infarctions, 1 terminal heart failure), shock following surgical treatment for intestinal ileus in 1 patient, and unknown cause in 2 patients. All deaths in patients with PAI-1 activity rise higher than 3.7 U/mL were of cardiovascular origin. Higher death rate was found in patients older than 65 (χ^2^ test, *P* = 0.034), women (χ^2^ test, *P* = 0.030), patients with heart failure (Fisher exact test, *P* = 0.026), worse final TIMI flow (Fisher-Halton-Freeman test, *P* = 0.001), lower left ventricular ejection fraction (LVEF) (Mann-Whitney U test, *P* = 0.014), and lower BMI (Mann-Whitney U test, *P* = 0.024).

**Figure 2:**
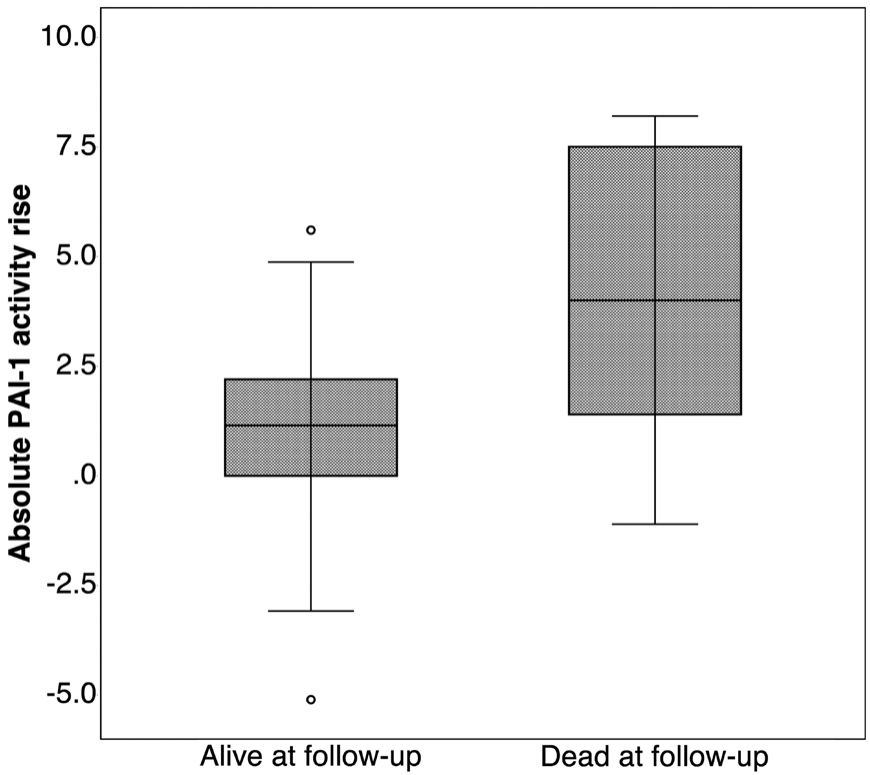
Plasminogen activator inhibitor-1 (PAI-1) activity rise according to patient status on follow-up (alive or dead). Boxes represent 25th and 75th percentile ranges, horizontal line represent medians, whiskers represent 1.5 times interquartile range, and circles represent outliers (patients with values higher than 1.5 times inter-quartile range).

**Figure 3:**
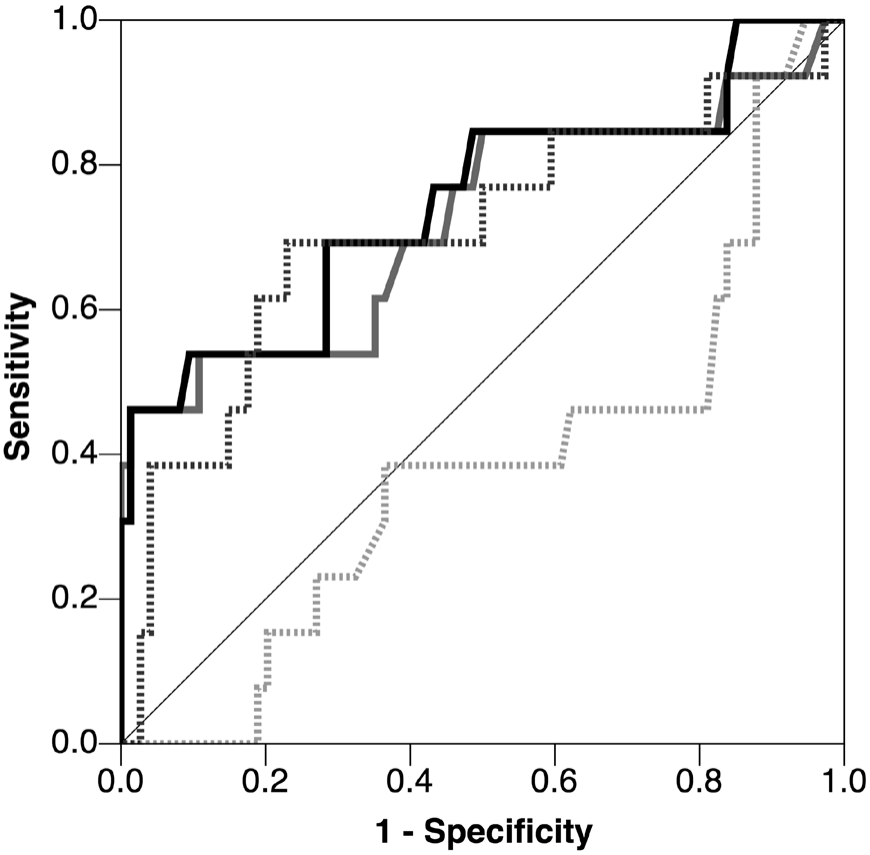
Receiver operating characteristic curve for plasminogen activator inhibitor-1 (PAI-1) activity (value on admission [dotted gray] and after 24 hours [full gray], absolute [full black] and percent rise [dotted black]) in relation to 5-year death rate.

**Figure 4:**
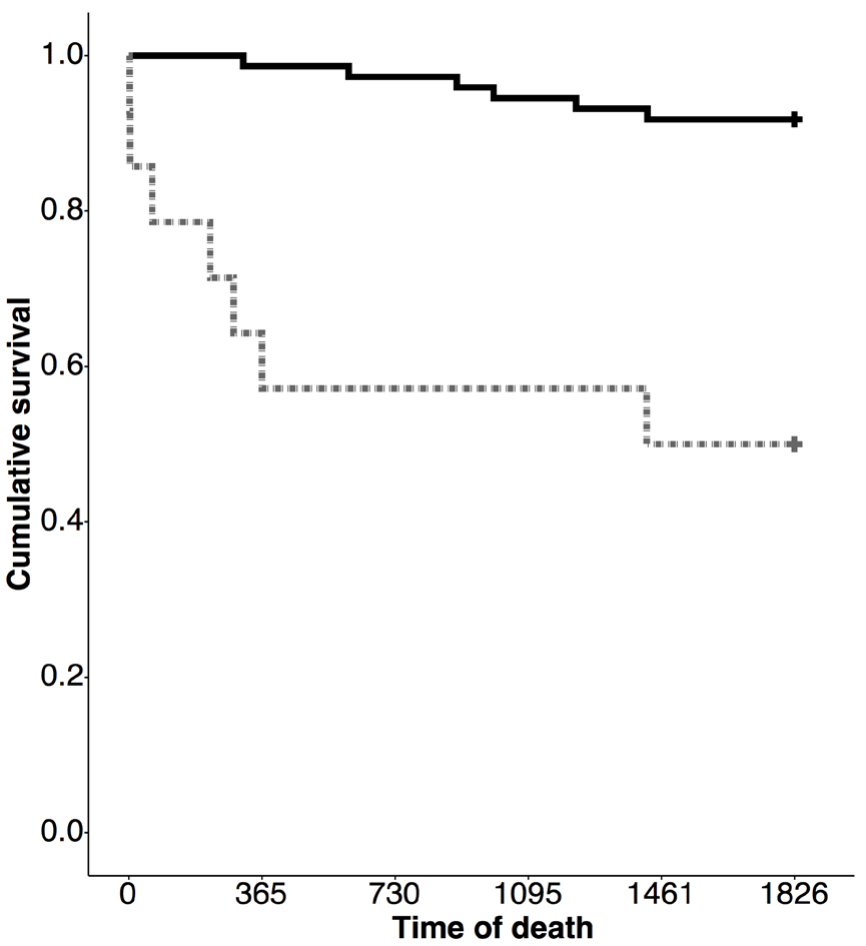
Kaplan-Meier survival curves according to plasminogen activator inhibitor-1 (PAI-1) rise (higher [gray line] or lower than 3.7 U/mL [black line]). Censored designated with cross.

PAI-1 activity rise was higher in patients deceased during follow-up (Figure 2), which was also observed for patients deceased during hospital treatment, as well as after discharge (Table 2). In ROC analyses (Figure 3), significance was reached for PAI-1 activity in the second sample and PAI-1 activity rise, with the largest area under the curve for PAI-1 activity rise (area under the curve 0.748, 95% CI 0.579-0.917) and the optimal cut-off point of 3.7 U/mL. Kaplan-Meier survival curves showed poorer 5-year outcome in patients with PAI-1 activity rise higher than 3.7 U/mL (χ^2^ = 22.7 and *P* < 0.001, Figure 4).

**Table 2:** Number of patients stratified by plasminogen activator inhibitor-1 (PAI-1) activity rise higher or lower than 3.7 U/mL and PAI-1 activity rise percentiles according to patient status at follow-up (alive or dead)

		No. of patients (% with PAI-1 rise)								
PAI-1 rise		total death rate			death during hospital stay			death during follow-up		
		alive	dead	*P*	alive	dead	*P*	alive	dead	*P*
>3.7 U/mL*	no	67 (92.0)	6 (8.0)	<0.001	73 (100.0)	0 (0)	0.024^†^	67 (92.0)	6 (8.0)	0.001
	yes	7 (50.0)	7 (50.0)		12 (86.0)	2 (14.0)		7 (58.0)	5 (42.0)	
Percentile^‡^	25th	0.00	1.40	<0.001	0.10	4.90	0.026	0.00	1.20	0.001
	50th	1.15	4.00		1.30	6.20^§^		1.15	2.19	
	75th	2.20	7.50		2.40	7.50		2.20	7.80	

*Cut-off values determined by receiver operating characteristic curve analysis regarding mortality. χ^2^ test was used if not stated otherwise.

†Fisher exact test.

‡Mann-Whitney U test.

§Median for only two cases.

Univariate Cox regression showed that PAI-1 activity rise higher than 3.7 U/mL was related to death at follow-up (hazard ratio 8.92, 95% CI 2.99-26.69, *P* < 0.001). Multivariate analysis using enter- and forward-stepwise approach included age, sex, heart failure occurrence, no-reflow phenomenon, thrombaspiration use, final TIMI flow (expressed as TIMI flow 3 or <3), and PAI-1 activity rise higher than 3.7 U/mL as covariates. In both approaches, the only independent predictors of death were final TIMI flow and PAI-1 activity rise higher than 3.7 U/mL (Table 3). After forcing LVEF into the model (using forward-stepwise approach), PAI-1 significance persisted despite 20 excluded patients due to missing LVEF values. PAI-1 and final TIMI flow remained significant after BMI, peak CK, multivessel disease detected on PCI, history of coronary artery disease, and diabetes were introduced into the model.

**Table 3:** Multivariate Cox regression with status variable of occurrence of death at 5-year follow-up. Cox regression model was significant with χ^2^ = 38.47 and *P* < 0.001

	Hazard ratio	*P*	95% confidence interval
Final thrombolysis in myocardial infarction flow <3	6.60	0.009	1.60-27.14
Plasminogen activator inhibitor-1 rise >3.7 U/mL	5.55	0.018	1.34-22.98
Occurrence of heart failure	2.48	0.203	0.61-10.02
No-reflow phenomenon	2.18	0.327	0.46-10.36
Female sex	1.30	0.700	0.34-4.99
Age	1.01	0.658	0.95-1.08
Thrombaspiration	0.96	0.957	0.20-4.50

## Discussion

We found that PAI-1 activity rise higher than 3.7 U/mL was associated with death during the 5-year follow-up in acute-phase STEMI patients treated with primary PCI. PAI-1 activity rise higher than 3.7 U/mL indicated poorer outcome, with Kaplan-Meier survival curves diverging within the first year after STEMI (suggesting the time frame in which PAI-1 activity rise exerts its impact), and the observed death rate of 50%. These results are in accordance with the study by Collet et al (7) on 30-day mortality of STEMI patients, in which the majority of deaths occurred during the hospital stay. However, the latter study included a heterogeneous patient population, a number of patients in the subacute MI phase, and had an incomplete penetration of invasive reperfusion strategy. Our study population comprised genuine STEMI patients with low initial TIMI flow levels, absence of initial TIMI 3 flow, substantial peak CK levels, and low initial troponin T levels in accordance with acute STEMI phase.

PAI-1 activity rise higher than 3.7 U/mL was associated with common high-risk features of STEMI, including heart failure, worse final TIMI flow, anterior myocardial wall infarction, left anterior descending artery as infarction-related artery, female sex, and higher peak CK levels. All these features except female sex are associated with a larger infarction size (11-13), which was estimated in our study by peak CK levels. Troponin T was used at recruitment only to rule in or rule out MI; therefore, consistent peak troponin T levels were not available for MI size estimation. Both heart failure and larger infarction size induce prominent inflammatory response in acute STEMI patients (14,15), while PAI-1 is one of the acute phase reactants (16). They might influence the outcomes separately, since PAI-1 activity rise was shown in multivariate Cox regression to be independently related to 5-year death rate. The only additional significant predictor of death in multivariate analysis was final TIMI flow, as was the case in the study by Collet et al (7). However, since no other predictors were significant, which might be the consequence of sample size, studies with larger sample size are needed to determine whether any additional variable is an independent predictor of death and whether PAI-1 is independent to these variables.

Higher plasma PAI-1 concentrations were detected in acute STEMI patients with fibrinolysis-resistant thrombi (17), while higher PAI-1 rise was detected in patients treated with streptokinase and persistent coronary artery occlusion (18). This indicates that PAI-1 rise influences the outcome similarly regardless of the reperfusion strategy. Enhanced PAI-1 activity may facilitate the persistence of microthrombi and a prothrombotic state in an early MI phase (19), which could worsen the short-term outcome in STEMI (7) and non-ST elevation acute coronary syndrome patients (20). On the other hand, PAI-1 plays a crucial role in myocardial fibrosis after MI, a fundamental component of unfavorable remodeling after STEMI and a possible pathway influencing the long-term outcome (21). PAI-1 deficiency protected against the induction of cardiac and perivascular fibrosis by a nitric oxide synthase inhibitor in an animal model (22). Yet, the complete PAI-1 absence in knockout murine models after artificially induced MI increased the incidence of myocardial rupture and death (23). Thus, both complete deficiency and high PAI-1 levels induced fibrosis in various organs, suggesting that the optimal therapeutic goal should be PAI-1 normalization (24). Accordingly, our results show that PAI-1 rise, rather than the initial PAI-1 activity, was related to a poorer long-term outcome. Hypothetically, PAI-1 rise could represent an individual phenotypic feature and could lead to a poorer outcome in subsequent cardiac or non-cardiac events after the patients survive the initial STEMI. Our data suggest that the influence of PAI-1 activity rise extends beyond the initial event. If future studies prove the causality of the observed association, PAI-1 antagonists use in MI treatment should be investigated. Currently, PAI-1 antagonists are still being developed, and no active substance is available for clinical use (25). Interestingly, in our study, concurrent medical therapy, including that with glycoprotein IIb/IIIa inhibitor (eptifibatide), did not influence PAI-1 activity rise. This is in accordance with previously published data showing deterred platelet aggregation and fibrin binding, but no difference in platelet PAI-1 secretion, after glycoprotein IIb/IIIa inhibition (26). Thus, even the most potent antiplatelet therapy did not affect the PAI-1 system.

PAI-1 activity values detected after 24 hours were significantly higher than admission values, which was not the case in the study by Collet et al (7), which analyzed PAI-1 antigen levels. Theoretically, PAI-1 activity reveals inhibitory capacity of PAI-1 system, while antigen levels cannot be used to assess PAI-1 protein status (active, inactive, or latent). This is why we believe that PAI-1 activity analyses are more appropriate for studying clinical entities with rapid changes in numerous biological systems, such as acute coronary syndromes.

At both measurement points, similar circadian rhythm was detected, with the highest values early in the morning. This pattern is described outside acute coronary syndrome (27) and is thought to be an evolutionary mechanism assuring higher blood clotting capability during physically most active part of the day. It is also responsible for peak MI occurrence in western societies (28). Our data suggest that intense events, such as acute STEMI, invasive treatment, and admission to the intensive care unit, do not affect the intrinsic circadian system in a short time period (24 hours), only consistently shift the PAI-1 time-activity curve upwards. Such circadian rhythm of PAI-1 variations in the acute phase of STEMI has not been previously described. Since single-sample PAI-1 activity determination cannot capture these variations in acute STEMI, we analyzed two samples 24 hours apart.

The major drawback of this study were the inadequate financial resources, which limited the sample size. However, the number of the included patients was not much smaller than in other PAI-1 studies in acute coronary syndromes (7,17,29,30). *Post-hoc* power analysis showed that χ^2^ and Mann-Whitney U tests for determining the difference in PAI-1 activities between dead and alive patients at 5-year follow-up had adequate power (1.000 for both tests at 5% significance level, G*Power 3.1.9.3, Heinrich-Heine-Universität Düsseldorf, Düsseldorf, Germany) (31). Furthermore, the enrolment period had started before ticagrelor and drug-eluting stents for primary PCI became widely available in Croatia. Although this is a prospective study, due to its observational design the results may not be generalized to a larger population.

In conclusion, PAI-1 activity rise in acute STEMI patients treated with primary PCI was associated with high-risk features and poorer long-term survival. This finding could improve the accuracy of long-term prognosis in STEMI patients. Further studies are needed to clarify whether the determined relation is casual or causative. If the latter is true, additional efforts to produce and clinically test PAI-1 antagonists are required.
